# Tissue engineering rib with the incorporation of biodegradable polymer cage and BMSCs/decalcified bone: an experimental study in a canine model

**DOI:** 10.1186/1749-8090-8-133

**Published:** 2013-05-20

**Authors:** Hua Tang, Bin Wu, Xiong Qin, Lu Zhang, Jim Kretlow, Zhifei Xu

**Affiliations:** 1 Department of Thoracic and Cardiovascular Surgery, Shanghai Changzheng Hospital, The Second Military Medical University, No.415 Fengyang Road, Shanghai 200003, China; 2 Shanghai Key Laboratory of Tissue Engineering, Shanghai 9th People’s Hospital, Shanghai Jiao Tong University School of Medicine, Shanghai, China; 3 Institute of Biosciences and Bioengineering, Rice University, Rice, USA

**Keywords:** Tissue engineering, Rib reconstruction, PDO, Long defect of bone

## Abstract

**Background:**

The reconstruction of large bone defects, including rib defects, remains a challenge for surgeons. In this study, we used biodegradable polydioxanone (PDO) cages to tissue engineer ribs for the reconstruction of 4cm-long costal defects.

**Methods:**

PDO sutures were used to weave 6cm long and 1cm diameter cages. Demineralized bone matrix (DBM) which is a xenograft was molded into cuboids and seeded with second passage bone marrow mesenchymal stem cells (BMSCs) that had been osteogenically induced. Two DBM cuboids seeded with BMSCs were put into the PDO cage and used to reconstruct the costal defects. Radiographic examination including 3D reconstruction, histologic examination and mechanical test was performed after 24 postoperative weeks.

**Results:**

All the experimental subjects survived. In all groups, the PDO cage had completely degraded after 24 weeks and been replaced by fibrous tissue. Better shape and radian were achieved in PDO cages filled with DBM and BMSCs than in the other two groups (cages alone, or cages filled with acellular DBM cuboids). When the repaired ribs were subjected to an outer force, the ribs in the PDO cage/DBMs/BMSCs group kept their original shape while ribs in the other two groups deformed. In the PDO cage/DBMs/BMSCs groups, we also observed bony union at all the construct interfaces while there was no bony union observed in the other two groups. This result was also confirmed by radiographic and histologic examination.

**Conclusions:**

This study demonstrates that biodegradable PDO cage in combination with two short BMSCs/DBM cuboids can repair large rib defects. The satisfactory repair rate suggests that this might be a feasible approach for large bone repair.

## Background

Rib defects are seen in many medical situations such as post excision of chest wall tumours [1,2], infection, necrosis [3], trauma and when part of a rib is used as the donor material to reconstruct other bone defects [4,5]. In the past, little attention was paid to rib defect reconstruction as it was always thought that to have little impact on respiratory function. With the development of improved surgical techniques and the increase of patient aesthetic concerns, rib reconstruction has gradually gained more attention. As rib defects are always large, to now there are few experimental reports on rib reconstruction.

Tissue engineering has been demonstrated to be a viable technique for regenerating large segments of bone [6,7]; however, few attempts have been made to tissue engineer ribs where a complete segmental defect exists.

When tissue engineering bone, two important factors must be considered chiefly among many others—seed cell and scaffold. Bone marrow mesenchymal stem cells (BMSCs) have repeatedly been demonstrated to be a suitable seed cell for bone tissue engineering [8-10]. As for the scaffold, significant research has been performed to identify the best material for bone tissue engineering. Autogenous bone is often considered to be the best scaffold for bone tissue engineering [8,11,12], but concerns over the limited ability and donor site morbidity limit its use in the treatment of large defects, so allograft and xenograft bone often become the first choice in clinical applications.

Polydioxanone (PDO), a synthetic resorbable polymer is now widely used as a suture material due to its strength and rate of degradation, but there are few reports about its use for other applications. Our previous work has included successful reconstruction of a chest wall defect spanning multiple ribs using a single PDO mesh [13].

For this study, we hypothesized that two 2-cm long DBM cuboids seeded with autogenous BMSCs could be placed with a 6-cm long PDO cage woven from PDO sutures and used to repair a 4-cm long single rib defect in the canine, proving the potential of reconstructing a single rib defect using multiple scaffolds seeded with BMSCs. We hypothesized that the PDO cage alone, or a PDO cage filled with two acellular cuboids would not equal the regenerative capability of the cell-seeded scaffolds.

## Methods

### Animals

Twelve mongrel dogs aged 1 to 2 years, weighing 12 to 15 kg, were used in this study. The 4^th^ and 7^th^ ribs of each dog were made defect. All the 7^th^ ribs were received PDO cage/DBMS/BMSCs, and six of all the 4^th^ ribs received PDO cages/DBM or PDO cages (Table 1). The experimental protocol was approved by the Animal Care and Experiment Committee of The Second Military Medical University.

**Table 1:** The grouping of the experimental dogs

	**Number of the animals**	**The reconstruction of 4th rib defect**	**The reconstruction of 7th rib defect**
group 1	6	no material	PDO cage/DBM/BMSCs
group 2	6	PDO cage/ DBM	PDO cage/DBM/BMSCs

### Preparation of DBMs/BMSCs

The demineralization process: The swine cancellous bone of the tibial plateau was prepared and washed with 50°C water repeatedly until it was clean. Then it would be dried up and degreased with Chloroform methanol under the 50°C temperature for 24 hours. The scaffold would be soaked in the H2O2 (volume fraction 300 ml/L) for another 24 hours. The degreasing process and soaking process would be repeated twice. Then the scafoolds would be dialyzed with double-distilled water for 12 hours. The last step is to deminerlized with 0.3 mol/L hydrochloric acid for 5 minutes and washed with clean water. The residual calcium was ranged from 12% to 20%. The pore size ranged from 150 um to 400 um.

**Figure 1:**
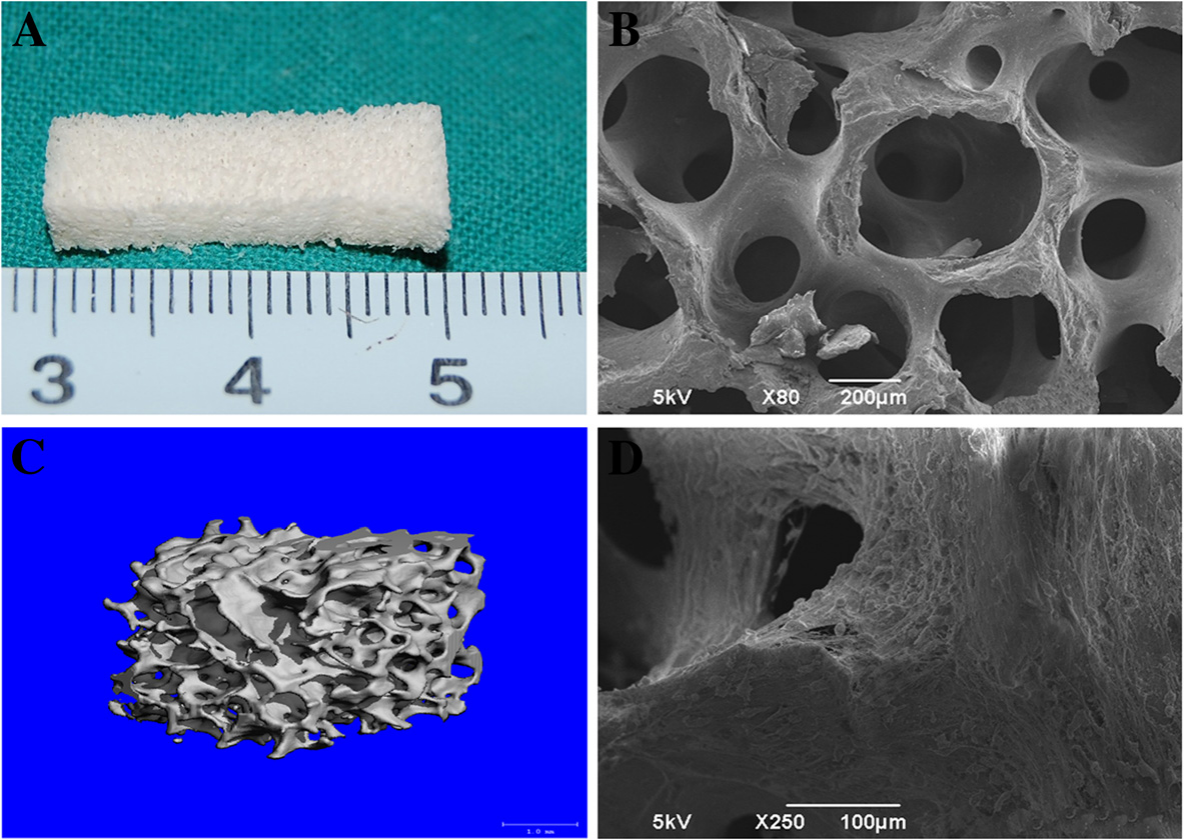
**The structure of DBM.** (**A**) shows the gross view of the DBM which was characterized by electron scanning microscope (**B**) (×80) and micro-CT (**C**). DBM has an interconnected porosity. (**D**)(×250) the microstructure of DBM/BMSCs. BMSCs are seen attaching on the wall of the DBM.

Swine demineralized bone matrix (Shanghai GuoRui Life Technology, Ltd., China) was molded into cuboids (20 × 10 × 5 mm) mimicking the normal structure of the rib (Figure 1A) with knife. Scanning electron microscopy (SEM, Figure 1B) and micro-CT (Figure 1C) were performed to characterize the DBM and showed that all pores of the cuboids were interconnected. Osteogenically induced BMSCs at passage 2 were harvested from the right or left iliac crest, collected, and resuspended in the osteogenic media (OM) consisting of Modified Eagle Medium (DMEM, Gibco, Grand Island, NY) supplemented with 10% fetal bovine serum (FBS, Gibco), 10^-8^ mol/L dexamethasone, 10 mmol/L β-phosphoglycerol and 50 mmol/L L-2-ascorbic acid (all from Sigma) at a density of 5 × 10^7^ cells/ml. When the cells reached 80–90% confluence, they were detached with 0.25% trypsin/EDTA (Gibco) and then subcultured at a density of 1 × 10^5^ cells/cm^2^ in 100-mm dishes. Cells at second passage were used in this study. Cells in suspension were slowly injected into the cuboids using a syringe (1 mL/cuboid). The DBMs/BMSCs were subsequently cultured for 7 days in vitro before implantation. In a parallel experiment, the same cuboids were prepared and seeded with BMSCs at an identical cell density. After 7 days incubation, the constructs were fixed in 2% formalin and examined with SEM (Figure 1D).

### Preparation of biodegradable polymer cage

**Figure 2:**
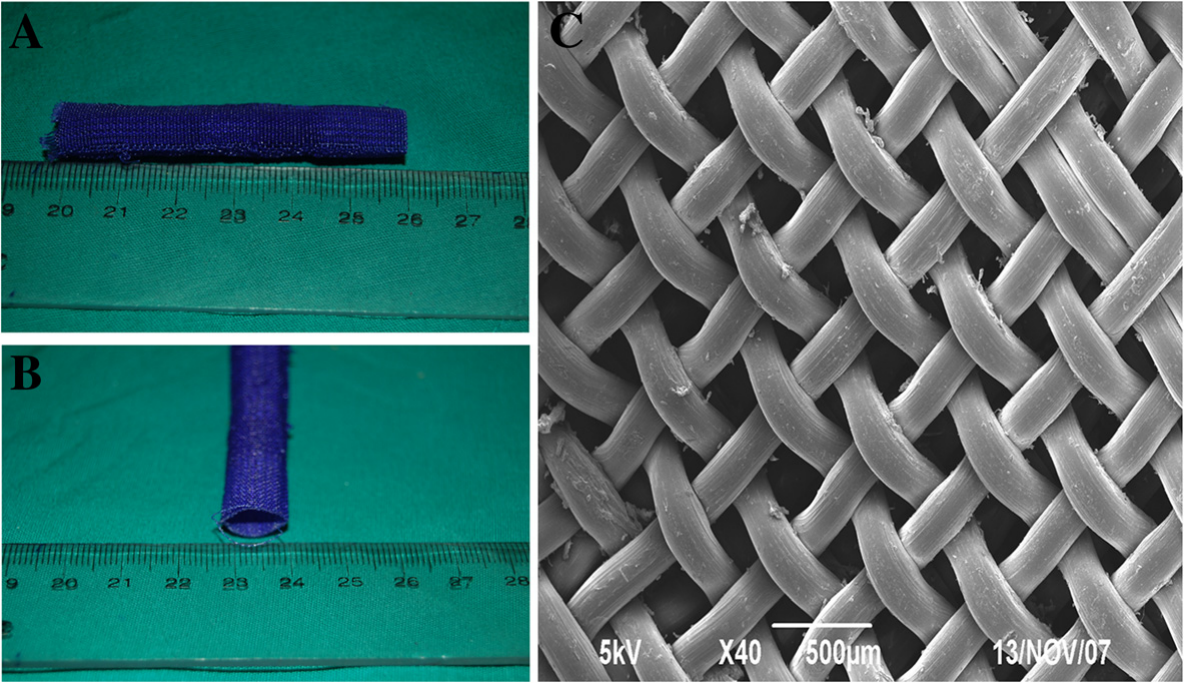
**Gross view and microstructure of the PDO cage.** The cage was 6cm in length and 1.2cm in diameter as shown in (**A**) and (**B**). Microstructure of the PDO cage (**C**) showed that has pores of size 250um × 250um which potentially allow the penetration of nutrients.

The biodegradable PDO thread with diameter 0.23 cm was prepared to construct the cage which is 6 cm in length and 1.2 cm in diameter by Donghua University. The cage can contain two 2 cm-long DBM cuboids and the remaining unfilled ends of the cage can overlap with the cut end of the partially resected rib that is to be repaired. The cage was also examined by scanning electron microscopy (Figure 2).

### Surgical procedures

**Figure 3:**
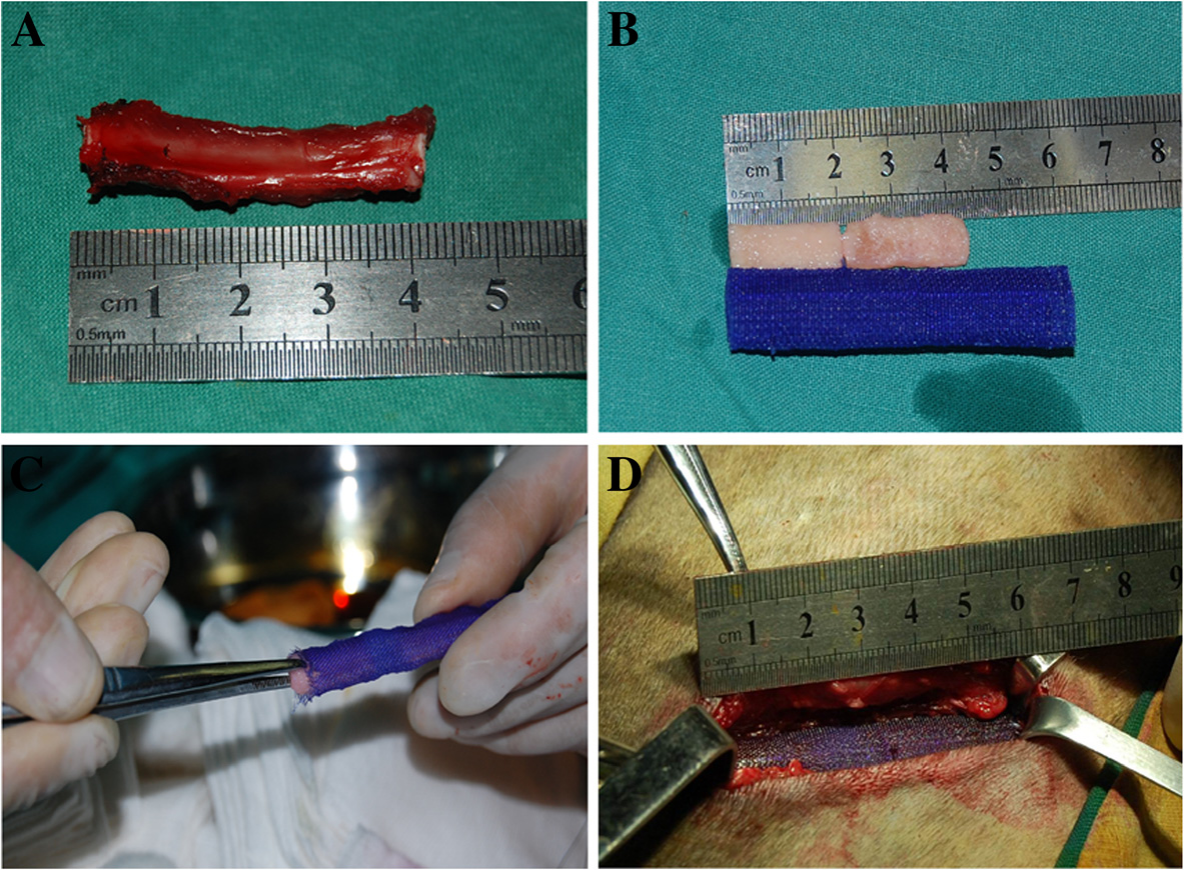
**The surgical procedure.** (**A**) the 4 cm long rib together with periosteum defect was created. (**B**) the preparation of DBMs/BMSCs and PDO cage. (**C**) The DBMs/BMSCs were put into the PDO cage. (**D**) The 6cm-long PDO cage containing two 2cm-long BMSCs/DBMs was then fit into the defect with the ends of the cage overlapping the cortex at both ends of the rib by 10 mm.

The dogs were operated on under general anesthesia with endotracheal intubation. Anesthesia was performed with intravenous sodium pentobarbital (30 mg/kg) throughout the procedure. The chest wall was randomly chosen as right or left. After anesthesia, the hair over the chest wall was shaved, and endotracheal intubation was performed before the dog was put in lateral position. The skin was sterilized with povidone iodine solution and wrapped using sterile technique. An incision was made parallel to the rib in the anterolateral region. A 4cm-long segmental defect of the seventh rib was created in the midportion together with periosteum on the surface of rib (Figure 3A). After completion of the resection, the PDO cage containing two 2 cm-long DBM/BMSCs (Figure 3B and Figure 3C) was then put into the defect with the ends of the cage overlapping the cortex at both ends of the rib by 10 mm (Figure 3D). The flank of the cage was sutured with the soft tissue around the rib to avoid surging with PDO suture. The same surgical procedure was performed in other groups. After surgery, prophylactic antibiotics (1,600,000 U of procaine penicillin and 80,000 U of gentamycin sulfate a day) were given immediately and maintained for 3 days.

### Radiographic examination

Three-dimensional (3D) reconstructions of the thoracic cage were accomplished using the Advantage Workstation 4.2 24 weeks after surgery to observe the regeneration of rib.

### Sample preparation and histological examination

The dogs were euthanized by means of an overdose of sodium pentobarbital at 24 weeks. The fourth and seventh ribs including defects were dissected. All dissected samples were photographed and then decalcified in 15% formic acid in formalin for 2–6 weeks. Tissue sections of samples were obtained for H&E staining. The costal tissue was also stained by H&E. The contralateral ribs were removed as normal controls.

### Mechanical test

The length of the samples was uniformly processed as 6 cm, including the reconstructed part. Each sample was tested using a three bending point test. The parameters of the test were as follows: L (test span) = 60 mm, load rate = 0.5N/mm, primary load = 1N. The bending stress was calculated using the equation: σ = 3PL/2bh2, where σ, P, L, b, and h represent the bending stress, the bending strength load, test span, the width and thickness of the specimen, respectively.

### Statistical analysis

Mechanical test data were analyzed by one-way ANOVA. The differences between the PDO cage/DBMs/BMSCs group (n = 12), PDO cage/DBMs group (n = 6), PDO cage group (n = 6) and normal rib group (n = 12) were assessed by Student–Newman–Keuls-q. The level of statistical significance was defined as p = 0.05.

## Results

### Gross observation

**Figure 4:**
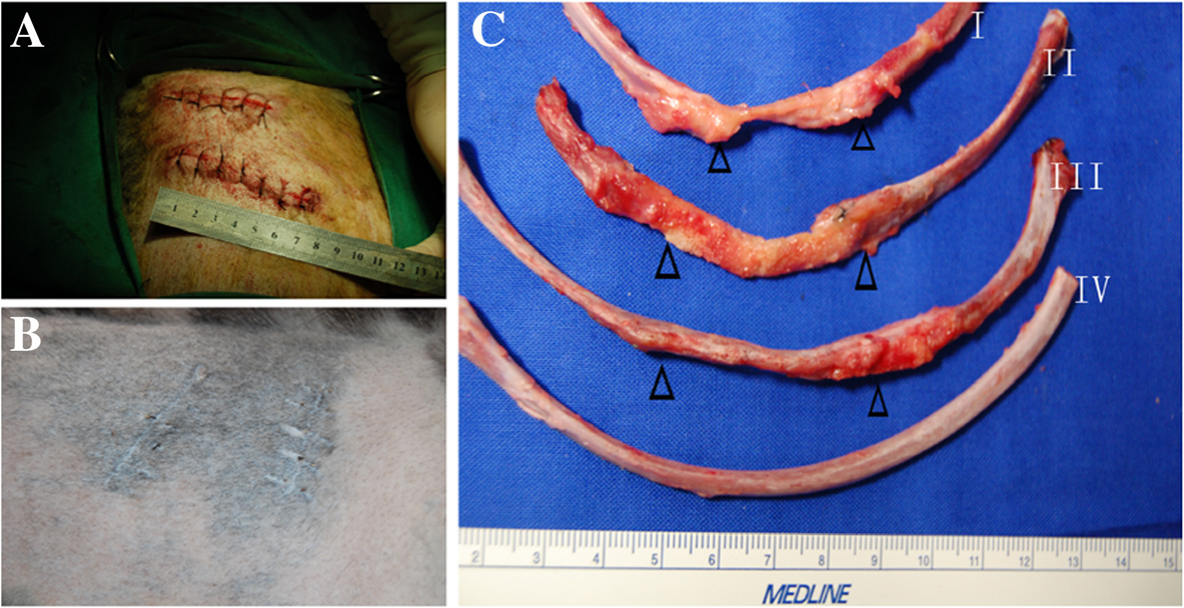
**The result of the experiment.** (**A**) and (**B**) show the situation of the wound 24 weeks after surgery; the wound healed well. (**C**) shows the gross view of reconstructed rib. (I) the flank group whose rib defect receives no material; (II) the control group whose rib defect received PDO cage/DBM; (III) the experimental group whose rib defect received PDO cage/DBM/BMSCs; (IV) the normal rib. The arrow shows the junction of the normal rib and scaffold.

All experimental dogs survived without any difficulty after surgery. No complications such as wound infection or paradoxical movement occurred after surgery. Better shape was achieved in the PDO cage/DBMs/BMSCs group than in the other two groups. When the repaired rib was subjected to an outer force, the ribs in the PDO cage/DBMs/BMSCs group kept their original shape while ribs in other two groups were easily deformed Additional file 1. In the PDO cage/DBMs/BMSCs group, we also observed bony union at all junctions. In the PDO cage/DBMs group, no bony union was observed not only in the junction of primary rib and scaffold but also in the junction of scaffold and scaffold; in the flank group, the defects between two cut ends of rib were filled with fibrous tissue (Figure 4; Additional file 2). In all groups, the PDO cage degraded completely.

### Radiographic examination

**Figure 5:**
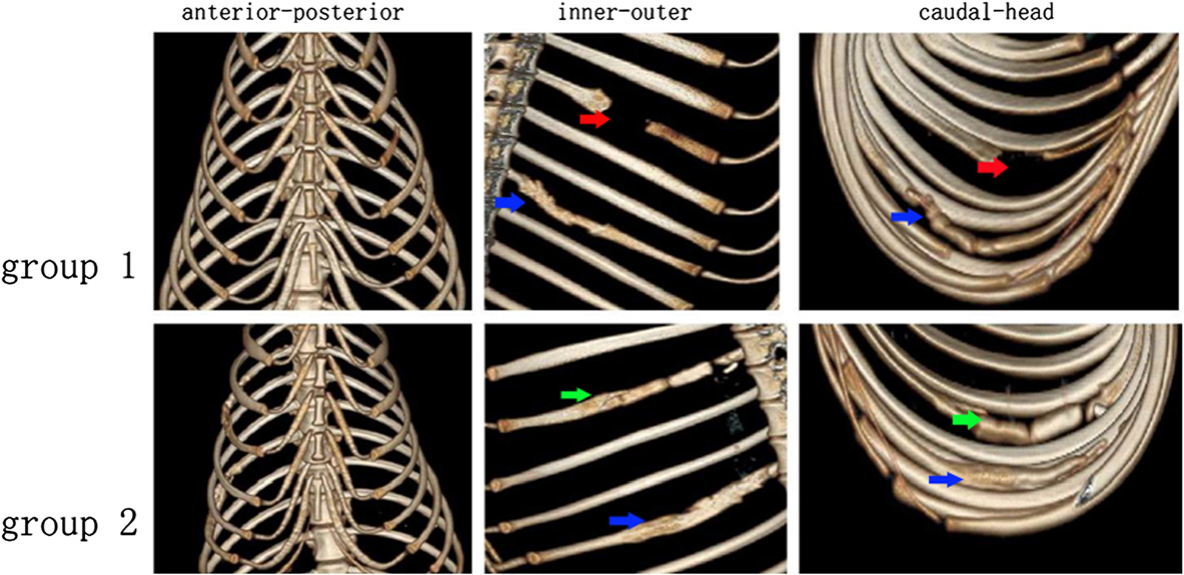
**3-D reconstruction of the thoracic cage.** We could see that there was no bone regeneration in the flank group, which is shown by the red arrow. In the PDO cage/DBMs group there was no new bone regeneration not only in the junction of two scaffolds but also in the junction between the rib and scaffold, which is shown as the green arrow. The radian of the reconstructed rib, however, was similar to the primary rib. In the PDO cage/DBMS/BMSCs, there was bone union in both the junctions which are shown with the blue arrow.

To observe new bone formation, CT-images were taken 24 weeks after surgery. Bony union was observed in the PDO cage/DBMs/BMSCs group while there was no union observed in the other two groups. Additionally, the reconstructed rib had an appropriate anatomic shape which correlated well to the primary radian (Figure 5).

### Histological examination

**Figure 6:**
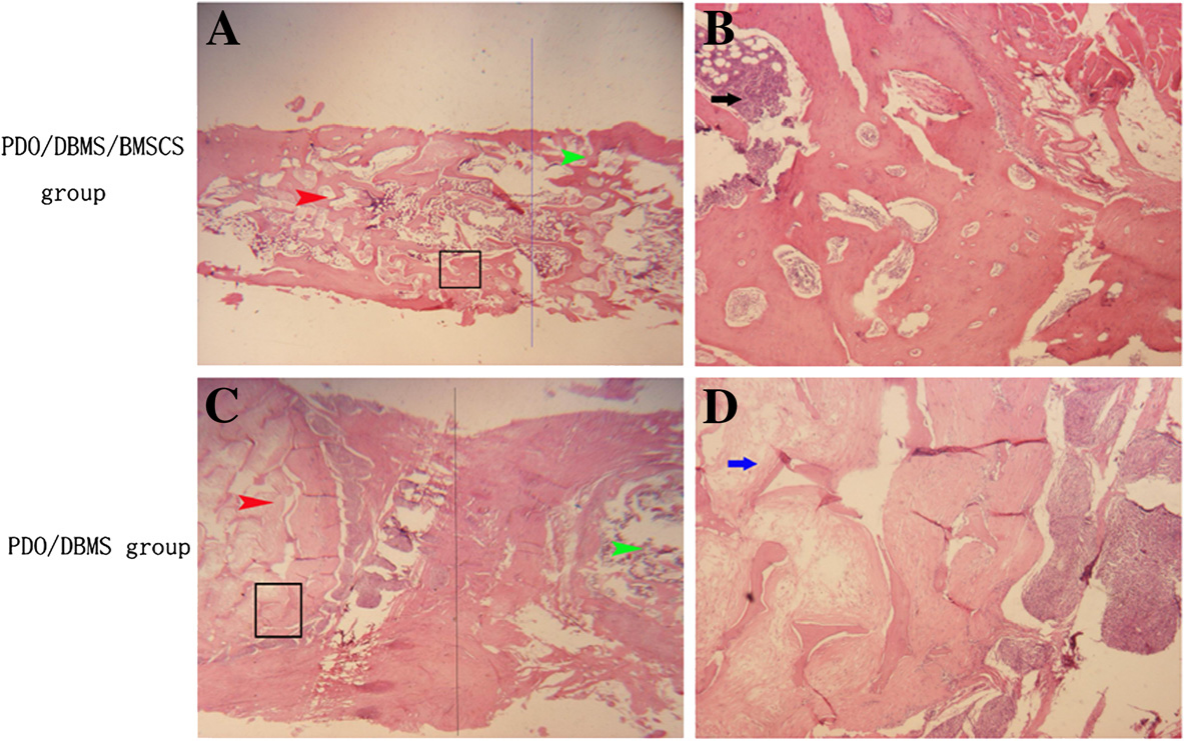
**H&E staining of the junction of primary rib (red triangular arrow) and scaffold (green triangular arrow).** (**A**) (×10) The primary rib and scaffold of the PDO/DBM/BMSCS group. No clear borderline was observed in the junction of the primary rib and scaffold. (**B**) (×40) At the junction of primary rib and scaffold of PDO/DBM/BMSCS group, marrow (black arrow) and new bone were observed; (**C**) (×10) In the primary rib and scaffold of the PDO/DBM group, a clear borderline was observed at the junction. (**D**) (×40) In the junction of the primary rib and scaffold of PDO/DBM group, fibrous tissue (blue arrow) was found both in the junction and in the scaffold.

**Figure 7:**
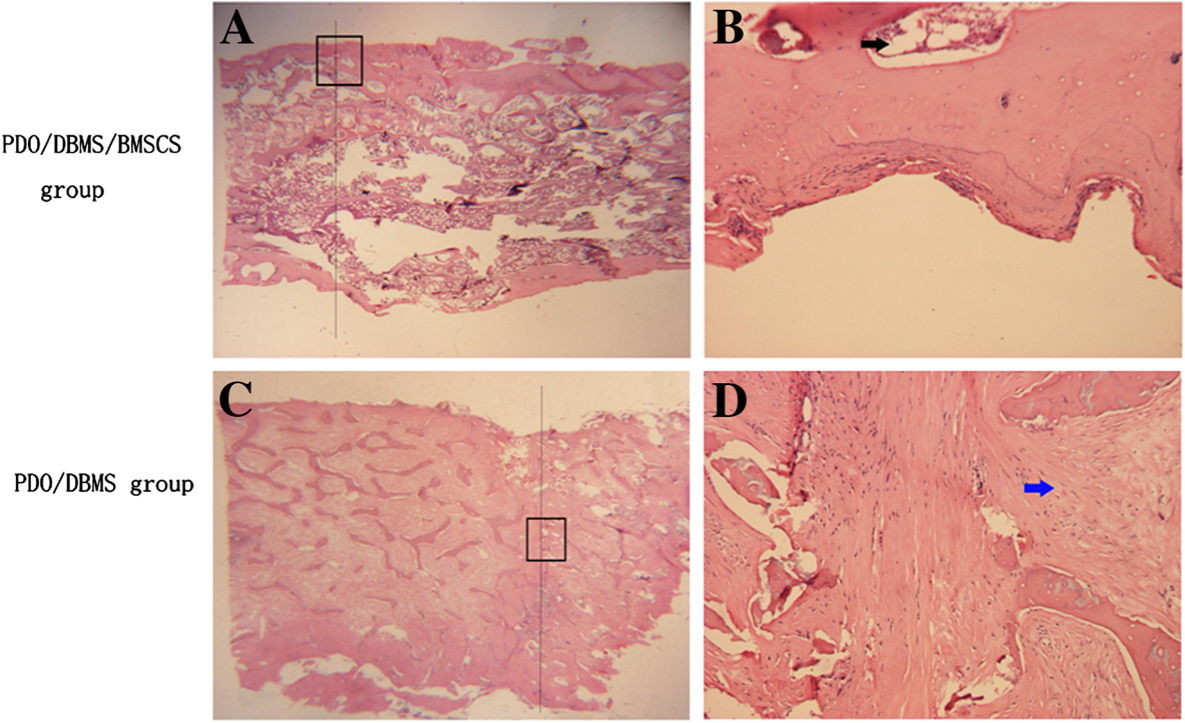
**H&E staining of the junction of scaffold and scaffold.** (**A**) (×10) the junction of two scaffolds of the PDO/DBM/BMSCS group. There was bone connection in the junction, and marrow was observed as is shown in Figure **B** (×100); (**C**) (×10) the junction of the two scaffolds of the PDO/DBM group. No bone connection was observed, and fibrous tissue (blue arrow) was seen in the junction and scaffold as is shown in figure (**D**) (×100).

H&E staining was performed to verify the regeneration of new bone. In the PDO cage/DBMs/BMSCs group, there was bony union both in the junction of two scaffolds and in the junction of the primary rib and scaffold (Figure 6). Marrow was also observed in the scaffold. In the PDO cage/DBMs group, there was fibrous tissue at the junction of scaffolds and the center of the scaffold (Figure 7). In the flank group, no new bone was found.

**Figure 8:**
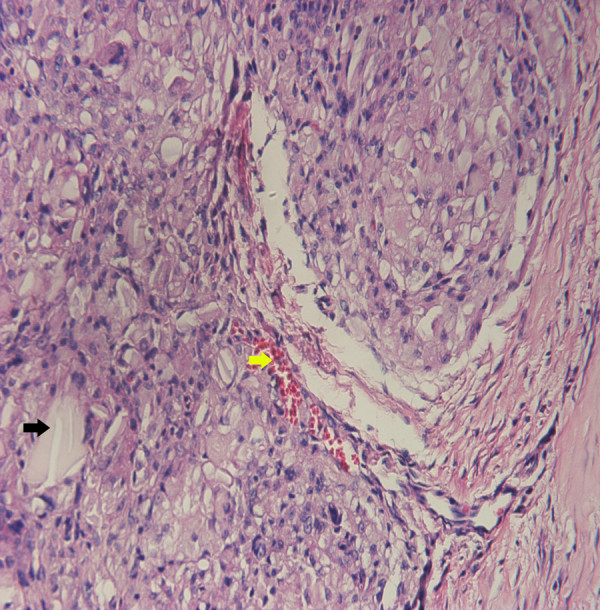
**H&E staining of the tissue around the reconstructed rib (×100).** The PDO degraded almost completely. Some pieces of PDO can be observed in the tissue and are shown by the black arrow. New blood vessels can be also seen in the tissue and are shown by the yellow arrow.

After 24 weeks, the PDO cages were almost completely degraded. Only some pieces of PDO were found in the tissue when examined by histology. Also, small vessels were observed in the tissue, which may help the penetration of nutrients (Figure 8).

### Mechanical test

To evaluate the mechanical properties of the reconstructed rib, three point bending tests were performed at 24 weeks after surgery. As there was no bone-union in the PDO cage group and the PDO cage/DBMs group, the three point bending test was not applied. In PDO cage/DBMs/BMSCs group (n = 12), the bending stress was 44.27 ± 2.31 Mpa, equivalent to 94.8% of the contralateral normal rib (46.67 ± 4.62 Mpa, n = 12). As there were only two groups, the assessment of the data was performed using a paired samples t-test. No significant difference were found between the two groups (P > 0.05).

## Discussion

The 4cm-long rib defect in this study meets the standard criteria of a critical size defect which is defined as a defect with length at least 2.5 times the diameter of the bone [14]. Also, when the defect was not repaired during surgery, after 24 postoperative weeks there was no evidence of bone regeneration in the defect. However, when this defect was reconstructed with the experimental construct, a biodegradable PDO cage and two BMSCs/decalcified bones, there was bone regeneration into the defect.

In this study, we tried to address two problems— fixation of scaffolds and repair of large bone defects. As for the first problem, two aspects including material and form have to be considered. As for the material for fixation, typically nondegradable materials, such as stainless steel or titanium [15,16], are deemed to be ideal as they have good mechanical properties which can maintain the stability of scaffolds. Such materials are now widely used in orthopedics for the fixation of various fractures. However, several problems still remain such as high cost, infection, difficulty molding the materials and the lack of degradation, which may result in many complications. Often, such materials have to be removed in an additional operation, adding to the patient’s pain and the cost associated with the initial operation. Furthermore, nondegradable materials are almost completely radiopaque, which obscures observation of the tissue around the material. Some imaging modalities, such as MRI, cannot be used when needed, which may delay the diagnosis of other diseases. Thus there is an increasing trend towards using degradable materials. In this study, we used PDO as the material of fixation. PDO has now been widely used for suture as it has excellent mechanical strength and degradability. It is reported that PDO sutures (PDS) can maintain their strength for about twenty weeks and will be completely resorbed after 180 days, which matches well to the speed of tissue regeneration [17,18]. In our study, we also found that the PDO cage was completely replaced by fibrous tissue which accords well with the result of other researchers’ experiments. As for degradation, hydrolysis into H_2_O and CO_2_ is the main method of degradation and should have little effect on the growth of BMSCs .

For the form of fixation, different methods such as chambers, cages and threads were used [19]. Porous cages are ideal for the fixation of scaffolds. First, the cage allows contact between the scaffold and primary rib, which can form an osseous connection in those junctions, adding to the stability of the rib. Second, the porous cage wall also allows for nutrient exchange with the material within the cage. The form of cage was first reported by Cobos et al. [20]. They repaired segmental long bone defects with cylindrical titanium cages. Over the next several years, this model was widely used. It is suitable for the reconstruction of load bearing bone as it has appropriate mechanical properties. The titanium cage, however, is nondegradable and radiopaque, which affects the observation of new bone and the tissue behind it. In this study, we utilized a PDO cage instead of a metal cage as the container for the scaffold. The PDO cage had appropriate degradability and is radiolucent, and thus did not affect the observation of organs in the thoracic cage. The PDO cage, however, had poor mechanical properties and can only be used in non-load bearing bones such as the rib, upper limb, etc. SEM characterization showed that the PDO cage also had a pores whose size is about 250 um × 250 um, sufficient to allow the penetration and exchange of nutrients and waste. Additionally, this cage is flexible and can match the radian of the chest wall.

The second problem is the repair of large bone defects, which was the critical part of our study. In the past several years, significant research directed towards addressing this problem, but little progress has been made. Tissue engineering is now an acknowledged technique for the reconstruction of bone defects, but, to date large bone defect repair remains a challenging problem. In the past, a single scaffold was widely used as a prosthetic for bone reconstruction or regeneration [21-23]. Some special considerations must be made when designing a scaffold to repair a rib defect. First, the rib has a variable radian and thus a single scaffold may not be suitable for rib reconstruction as the scaffold must be easily molded. In this study, we used multiple pieces of scaffold to reconstruct one bone defect. This method was first reported by Masatoshi et al. [24]. They successfully reconstructed a long rib defect with sixteen small, porous TCP scaffolds connected with titanum wire and covered with periosteum.

There still, however, remain several problems such as the mechanical integrity of the scaffolds. Although rib is a bone that suffers very little outer force, because of the effect of the chest muscle, the scaffold still is exposed to deforming forces, especially when the animal vocalizes. In the present study, we used DBM as the scaffold due to the suitable mechanical properties and porosity, which seemed to make it a more ideal scaffold than tricalcium phosphate. First, DBM has good osteoconductivity, osteoinductivity and osteogenic potential and has been widely used in orthopedic applications. Second, the rib is not a load bearing bone but may move with respiration. Thus we need a firm fixation of the scaffold. Third, the cortex of bone is the main component of the rib and yields very few seed cells for native tissue regeneration. Thus, a tissue engineering approach which can supply seed cells is the most suitable method for rib reconstruction.

Although we have achieved a good result with respect to the fixation of the scaffold and rib reconstruction, some problems should be studied further. First, the PDO cage as the material for fixation can only be used in non load bearing bone. Other materials or a method to augment the mechanical strength of the PDO should be investigated. Second, attempts should be made to optimize the bone regeneration. Nutrients are important to the seed cells, but the scaffolds used do not allow enough nutrients to reach cells seeded at the center of the scaffold. Thus it will be necessary to optimize the scaffold size and configuration with regard to the resultant mechanical properties so that a stable scaffold that allows maximal nutrient transfer is used. Third, xenograft may have rejection reaction if it is not properly managed and thus it may have worse effect of bone regeneration compared to autograft. Fourth, much research is needed into the seed cell source and any necessary manipulations that may be performed prior to implantation such as cell expansion or transformation. A number of options are currently being investigated, and the ideal seed cell for bone tissue engineering has not been identified.

## Conclusion

In our study, we successfully reconstructed large rib defects with biodegradable PDO cages in combination with two short DBM/BMSCs constructs. New bone regeneration was verified not only between the two scaffolds but also in the junction of scaffold and primary rib. We think that such a technique might be a feasible approach for large bone repair but further research should be done.

## Abbreviations

PDO: Polydioxanone; DBM: Demineralized bone matrix; BMSCs: Bone marrow mesenchymal stem cells; 3D: Three-dimensional; SEM: Scanning Electron Microscope.

## Competing interests

The authors declare that they have no competing interests.

## Authors’ contributions

HT designed the study, carried out animal study, followed up the animals and drafted the manuscript. BW performed the operation of the animal. XQ participated in the design of the study and performed the statistical analysis. LZ conceived of the study, and participated in its design. KJD participated in the design and helped to draft the manuscript. ZX designed the study, carried out animal study and rectified the manuscript. All authors read and approved the final manuscript.

## Supplementary Material

Additional file 1 The normal rib. Click here for file

Additional file 2 The rib defect was reconstructed with PDO cage/DBM. Click here for file

## References

[B1] Gupta SS Singh O Soni M Raikwar RS Mathur RK Extra-osseous Ewing's sarcoma of chest wall ANZ J Surg 2009 79 10 752 753 19878174 10.1111/j.1445-2197.2009.05063.x

[B2] Schwartz GS Rios L Zivin-Tutela T Bhora FY Connery CP Uncommon etiology of an anterior chest wall mass Ann Thorac Surg 2009 88 5 e58 e59 10.1016/j.athoracsur.2009.07.090 19853079

[B3] Hidalgo DA Saldana EF Rusch VW Free flap chest wall reconstruction for recurrent breast cancer and radiation ulcers Ann Plast Surg 1993 30 4 375 380 10.1097/00000637-199304000-00017 8512298

[B4] Kridel RW Ashoori F Liu ES Hart CG Long-term use and follow-up of irradiated homologous costal cartilage grafts in the nose Arch Facial Plast Surg 2009 11 6 378 394 10.1001/archfacial.2009.91 19917899

[B5] Bapat MR Chaudhary K Garg H Laheri V Reconstruction of large iliac crest defects after graft harvest using autogenous rib graft: a prospective controlled study Spine 2008 33 23 2570 2575 10.1097/BRS.0b013e318185287d 18978597

[B6] Jie Y Lei C Wen Jie Z Yilin C Repair of canine mandibular bone defects with bone marrow stromal cells and porous β-tricalcium phosphate Biomaterials 2007 28 6 1005 1013 10.1016/j.biomaterials.2006.10.015 17092556

[B7] Garreta E Gasset D Semino C Fabrication of a three-dimensional nanostructured biomaterial for tissue engineering of bone Biomol Eng 2007 24 1 75 80 10.1016/j.bioeng.2006.05.017 16846750

[B8] Syed-Picard FN Larkin LM Shaw CM Arruda EM Three-dimensional engineered bone from bone marrow stromal cells and their autogenous extracellular matrix Tissue Eng Part A 2009 15 1 187 195 10.1089/ten.tea.2007.0140 18759662

[B9] Zhou Y Chen F Ho ST Woodruff MA Lim TM Hutmacher DW Combined marrow stromal cell-sheet techniques and high-strength biodegradable composite scaffolds for engineered functional bone grafts Biomaterials 2007 28 5 814 824 10.1016/j.biomaterials.2006.09.032 17045643

[B10] Shao X Goh JC Hutmacher DW Lee EH Zigang G Repair of large articular osteochondral defects using hybrid scaffolds and bone marrow-derived mesenchymal stem cells in a rabbit model Tissue Eng 2006 12 6 1539 1551 10.1089/ten.2006.12.1539 16846350

[B11] Barone A Covani U Maxillary alveolar ridge reconstruction with nonvascularized autogenous block bone: clinical results J Oral Maxillofac Surg 2007 65 10 2039 2046 10.1016/j.joms.2007.05.017 17884536

[B12] Da Silva RV Bertran CA Kawachi EY Camilli JA Repair of cranial bone defects with calcium phosphate ceramic implant or autogenous bone graft J Craniofac Surg 2007 18 2 281 286 10.1097/scs.0b013e31802d8ac4 17414276

[B13] Qin X Tang H Zhifei X Zhao X Sun Y Gong Z Chest wall reconstruction with two types of biodegradable polymer prostheses in dogs Eur J Cardiothorac Surg 2008 34 4 870 874 10.1016/j.ejcts.2008.06.038 18678508

[B14] Lindsey RW Probe R Miclau T Alexander JW Perren SM The effects of antibiotic-impregnated autogenic cancellous bone graft on bone healing Clin Orthop Relat Res 1993 291 303 312 8504612

[B15] Segal N Hell J Berzins DW Influence of stress and phase on corrosion of a superelastic nickel-titanium orthodontic wire Am J Orthod Dentofacial Orthop 2009 135 6 764 770 10.1016/j.ajodo.2007.04.042 19524836

[B16] Hamad AM Marulli G Bulf R Rea F Titanium plates support for chest wall reconstruction with Gore-Tex dual mesh after sternochondral resection Eur J Cardiothorac Surg 2009 36 4 779 780 10.1016/j.ejcts.2009.04.047 19520586

[B17] Mäkelä P Pohjonen T Törmälä P Waris T Ashammakhi N Strength retention properties of self-reinforced poly L-lactide (SR-PLLA) sutures compared with polyglyconate (Maxon) and polydioxanone (PDS) sutures: an in vitro study Biomaterials 2002 23 12 2587 2592 10.1016/S0142-9612(01)00396-9 12033607

[B18] Kontio R Ruuttila P Lindroos L Suuronen R Salo A Lindqvist C Biodegradable polydioxanone and poly(l/d)lactide implants: an experimental study on peri-implant tissue response Int J Oral Maxillofac Surg 2005 34 7 766 776 10.1016/j.ijom.2005.04.027 15979853

[B19] Lindsey RW Gugala Z Milne E Sun M Gannon FH Latta LL The efficacy of cylindrical titanium mesh cage for the reconstruction of a critical-size canine segmental femoral diaphyseal defect J Orthop Res 2006 24 7 1438 1453 10.1002/jor.20154 16732617

[B20] Cobos J Lindsey RW Gugala Z The cylindrical titanium mesh cage for the treatment of a long segmental bone defect: description of a new technique and report of two cases J Orthop Trauma 2000 14 1 54 59 10.1097/00005131-200001000-00011 10630804

[B21] Dallari D Fini M Stagni C Torricelli P Nicoli Aldini N Giavaresi G In vivo study on the healing of bone defects treated with bone marrow stromal cells, platelet-rich plasma, and freeze-dried bone allografts, alone and in combination J Orthop Res 2006 24 5 877 888 10.1002/jor.20112 16609976

[B22] Shao XX Hutmacher DW Ho ST Goh JC Lee EH Evaluation of a hybrid scaffold/cell construct in repair of high-load-bearing osteochondral defects in rabbits Biomaterials 2006 27 7 1071 1080 10.1016/j.biomaterials.2005.07.040 16129483

[B23] Chistolini P Ruspantini I Bianco P Corsi A Cancedda R Quarto R Biomechanical evaluation of cell-loaded and cell-free hydroxyapatite implants for the reconstruction of segmental bone defects J Mater Sci Mater Med 1999 10 12 739 742 10.1023/A:1008939524807 15347943

[B24] Hoshino M Egi T Terai H Namikawa T Takaoka K Regenerative repair of long intercalated rib defects using porous cylinders of β-Tricalcium phosphate: an experimental study in a canine model Plast Reconstr Surg 2007 119 5 1431 1439 10.1097/01.prs.0000256319.89619.c8 17415237

